# Shared Decision-Making in Cosmetic Procedures and Aesthetic Plastic Surgery: A Systematic Review of Decision Aids, Clinical Benefits, Ethical Challenges, and Future Perspectives

**DOI:** 10.3390/healthcare14213672

**Published:** 2026-10-29

**Authors:** Dimitra Daskalopoulou, Spyridon A. Vourtsis

**Affiliations:** 1 Department of Anatomy, School of Medicine, Faculty of Health Sciences, National and Kapodistrian University of Athens, 75 Mikras Asias str., Goudi, 11527 Athens, Greece; 2 Department of Plastic Surgery, 417 Army Equity Fund Hospital, 10-12 Monis Petraki str., 11521 Athens, Greece; vourtsis@plastikos.eu

**Keywords:** shared decision-making, aesthetic plastic surgery, decision aids, artificial intelligence, patient-centered care

## Abstract

Background/Objectives: Shared decision-making (SDM) is essential to patient-centered care in aesthetic plastic surgery, where successful outcomes depend on aligning interventions with patients’ values, preferences, and goals. Decision aids may traditionally support SDM by presenting information in a structured manner and facilitating patient–clinician collaboration, while the emergence of artificial intelligence (AI) may further support SDM through medical information delivery and patient counseling. Methods: A systematic search of PubMed, Cochrane Library, and Google Scholar identified English-language studies published between 2011 and 2026 examining the applicability and impact of SDM as well as interventions, tools, or technologies that may influence the decision-making process, relevant to preference elicitation, patient education, and risk communication in aesthetic plastic surgery and cosmetic procedures. Two reviewers independently screened records and full texts, with disagreements resolved by discussion. Risk of bias was assessed using appropriate frameworks. Limitations, ethical considerations, and future perspectives were also evaluated. Results: Of 5485 records identified, 108 underwent full-text evaluation, and 38 met the inclusion criteria. Decision-support strategies were variably associated with greater patient participation, understanding, satisfaction, and enhanced expectation management. However, standardized, validated decision aids remain limited, and substantial methodological heterogeneity and moderate-to-high risk of bias restrict the strength of available evidence. Studies exploring AI-based technologies evaluated mainly feasibility and accuracy, while limited evidence was available concerning patient satisfaction, improvement in SDM, or clinical outcomes. Conclusions: The use of standardized decision aids across aesthetic plastic surgery remains limited, while the adoption of AI in SDM should be considered as an adjunct to human counseling. Prospective comparable studies are required to determine their effectiveness in a clinical framework.

## 1. Introduction

Healthcare, an aggregate of activities aiming to restore and maintain human well-being, is continually evolving by embracing patients’ values, preferences, and experiences into the process of decision-making and placing the patient at the center of its cosmos.

Shared decision-making (SDM) is a healthcare approach in which physicians and patients work collaboratively in the context of bidirectional communication to make decisions based on the best available evidence and clinical expertise, incorporating, concurrently, the patients’ informed values and preferences [1,2]. This collaborative process, in which clinicians guide patients by communicating the available treatment options accompanied by their potential benefits and risks, and based on the patient’s individual perspective and goals, has shown potential in patient care by inducing higher levels of patient satisfaction [1].

Traditionally, informed consent has constituted a fundamental component of patient care [3]. The introduction of SDM in the interaction between physician and patient has enabled the active participation of patients by communicating their values and preferences, extending beyond disclosure and consent [2]. Decision aids constitute tools that may assist the process of SDM by presenting information about the situation concerning the patients as well as the available options accompanied by their anticipated outcomes in a comprehensive and structured way [2,4].

The discipline of aesthetic plastic surgery increasingly implicates patients who are expressing even more immense demands, and as a result, an amplifying need for reliable and evidence-based approaches has emerged in order to be able to apply and reinforce informed decision-making [5]. Aesthetic plastic surgeons frequently encounter clinical dilemmas in which no singular treatment pathway is clearly the most suitable. Hence, the most pertinent management strategy entails a comprehensive decision-making pattern [6].

Artificial intelligence (AI) is increasingly being investigated in the provision of healthcare regarding patient education, outcome visualization, risk assessment, and alignment of shared decision-making with the growing demands of patients and society [7]. In aesthetic practice, its technological domains may support patient counseling by means of assessing the individual characteristics, matching the relevant objective data with available options, and simulating potential outcomes relevant to each option. Concurrently, AI applications may contribute to the concept of patient education by providing procedure-related information and guidance, and potentially reinforce the process of shared decision-making by incorporating technological guidance into human counseling and treatment planning. Demonstrated improvement in SDM or clinical outcomes, however, remains to be established [7].

The aim of this systematic review was to evaluate the implementation of SDM strategies and decision aids in aesthetic plastic surgery and cosmetic procedures and explore their contribution to the process of decision-making, as well as relevant limitations, ethical considerations, and future perspectives.

## 2. Materials and Methods

A systematic review with a narrative synthesis was undertaken to evaluate and comprehensively explore the impact of SDM strategies and decision-support methods in aesthetic plastic surgery and cosmetic procedures. The review methodology was developed in accordance with the Preferred Reporting Items for Systematic Reviews and Meta-Analyses (PRISMA) guidelines, where applicable, given the substantial heterogeneity of the available evidence.

### 2.1. Study Design and Selection Process

We conducted an extensive literature search to identify relevant studies using the electronic databases of PubMed, Cochrane Library, and Google Scholar, restricting our search to articles published across the time interval of 2011 and 2026 in the English language. PubMed was last searched on 4 August 2026, while the databases of Cochrane Library and Google Scholar were last searched on 14 September 2026. In conjunction with the initial search, the reference lists of the included studies and relevant review articles were manually examined to identify additional eligible publications in order to maximize the completeness of the review. The search strategy included terms for shared decision-making, decision aids, decision-support strategies, as well as digital and AI-based technologies in conjunction with aesthetic plastic surgery and cosmetic, non-surgical procedures.

A combination of keywords and Boolean operators was employed with regard to the literature search in PubMed: (“shared decision making”[tiab] OR “digital tool”[tiab] OR “patient-reported outcome measures”[tiab] OR “skin analysis”[tiab] OR “facial analysis”[tiab] OR “skin analyzer”[tiab] OR “simulator software”[tiab] OR “surgery simulator”[tiab] OR “3D imaging”[tiab] OR “3D simulation”[tiab] OR “teleconsultation”[tiab] OR “telemedicine”[tiab] OR “3-D simulation”[tiab] OR “3-D imaging”[tiab] OR “three-dimensional imaging”[tiab] OR “virtual reality”[tiab] OR “mobile application”[tiab] OR “augmented reality”[tiab] OR “decision support system”[tiab] OR “machine learning”[tiab] OR “ChatGPT”[tiab] OR “informed consent”[tiab] OR “decision Aid”[tiab] OR “artificial intelligence”[tiab] OR “decision tool”[tiab] OR “shared decision-making”[tiab]) AND (mammoplasty[tiab] OR mammaplasty[tiab] OR “breast implants”[tiab] OR “aesthetic surgery”[tiab] OR “breast augmentation”[tiab] OR “breast reduction”[tiab] OR “mastopexy” [tiab] OR “cosmetic surgery”[tiab] OR “abdominoplasty”[tiab] OR “blepharoplasty”[tiab] OR facelift[tiab] OR “body contouring”[tiab] OR “Hair Transplantation”[tiab] OR Injectables[tiab] OR “Laser Skin Resurfacing”[tiab] OR liposuction[tiab] OR rhinoplasty[tiab] OR “oculoplastic surgery”[tiab] OR “otoplasty”[tiab] OR “plastic surgery”[tiab] OR “gynecomastia”[tiab] OR “rhytidectomy”[tiab] OR “brow Lift”[tiab] OR “lipoplasty”[tiab] OR “tummy tuck”[tiab] OR “brachioplasty”[tiab] OR “arm lift”[tiab] OR “thigh lift”[tiab] OR “mummy makeover”[tiab] OR “skin rejuvenation”[tiab] OR “aesthetic medicine”[tiab] OR “cosmetic medicine”[tiab] OR “aesthetic plastic surgery”[tiab] OR “facial plastic surgery”[tiab] OR “cosmetic procedures”[tiab] OR “aesthetic procedures”[tiab] OR “non-surgical aesthetic treatments”[tiab] OR “aesthetic treatments”[tiab] OR “genioplasty”[tiab] OR “neck lift”[tiab] OR “lip augmentation”[tiab] OR “gluteal augmentation”[tiab] OR “buttock augmentation”[tiab] OR “fat grafting”[tiab] OR “autologous fat transfer”[tiab] OR “botulinum toxin”[tiab] OR “hyaluronic acid”[tiab] OR “biostimulators”[tiab] OR “dermal fillers”[tiab] OR “facial rejuvenation”[tiab] OR “injectable treatments”[tiab] OR “laser resurfacing”[tiab] OR “thread lifting”[tiab]).

A similar combination of terms was applied during the literature search in the Google Scholar database: (“shared decision making” OR “decision aid” OR “decision tool” OR “decision support”) (“artificial intelligence” OR “machine learning” OR ChatGPT OR “3D simulation” OR “3D imaging” OR “digital tool”) (“aesthetic procedure” OR “aesthetic surgery” OR “cosmetic surgery” OR rhinoplasty OR mammoplasty OR “breast augmentation” OR “abdominoplasty” OR “facelift”).

The Cochrane Library search additionally merged MeSH (Medical Subject Headings) descriptors for Decision Making. Shared; Decision Support Techniques; Artificial Intelligence; and Decision Making with a combination of relevant keywords: (“shared decision making” OR “shared decision-making” OR “digital tool” OR “patient-reported outcome measures” OR “skin analysis” OR “facial analysis” OR “skin analyzer” OR “simulator software” OR “surgery simulator” OR “3D imaging” OR “3D simulation” OR “3-D simulation” OR “3-D imaging” OR “three-dimensional imaging” OR teleconsultation OR telemedicine OR “virtual reality” OR “mobile application” OR “augmented reality” OR “decision support system” OR “machine learning” OR ChatGPT OR “informed consent” OR “decision aid” OR “artificial intelligence” OR “decision tool”) AND (mammoplasty OR mammaplasty OR “breast implants” OR “aesthetic surgery” OR “breast augmentation” OR “breast reduction” OR mastopexy OR “cosmetic surgery” OR abdominoplasty OR blepharoplasty OR facelift OR “body contouring” OR “hair transplantation” OR injectables OR “laser skin resurfacing” OR liposuction OR rhinoplasty OR “oculoplastic surgery” OR otoplasty OR “plastic surgery” OR gynecomastia OR rhytidectomy OR “brow lift” OR lipoplasty OR “tummy tuck” OR brachioplasty OR “arm lift” OR “thigh lift” OR “mummy makeover” OR “skin rejuvenation” OR “aesthetic medicine” OR “cosmetic medicine” OR “aesthetic plastic surgery” OR “facial plastic surgery” OR “cosmetic procedures” OR “aesthetic procedures” OR “non-surgical aesthetic treatments” OR “aesthetic treatments” OR genioplasty OR “neck lift” OR “lip augmentation” OR “gluteal augmentation” OR “buttock augmentation” OR “fat grafting” OR “autologous fat transfer” OR “botulinum toxin” OR “hyaluronic acid” OR biostimulators OR “dermal fillers” OR “facial rejuvenation” OR “injectable treatments” OR “laser resurfacing” OR “thread lifting”).

Titles and abstracts of the identified studies were evaluated in consonance with the predefined eligibility criteria outlined below. Two reviewers independently conducted study selection, and disagreements were discussed and resolved through collaborative review and consensus.

### 2.2. Eligibility Criteria

Studies were considered eligible if they incorporated the use of decision aids and interventional tools such as digital and AI technologies, 3D simulators, predictive models, virtual and augmented reality, and large language model applications with the role of facilitating patient counseling, risk communication, expectation management, and preference elicitation, and if they could, thus, improve shared decision-making in aesthetic plastic surgery and cosmetic procedures. Studies were included if they focused on an intervention or decision aid that could facilitate SDM during consultation or assessed digital technologies and artificial intelligence that could influence patients’ decision-making process, given the current rise in technology-assisted patient support. However, technical studies were not considered equivalent evidence of clinical effectiveness. Patient-reported outcome measures (PROMs) were considered separately from formal decision aids and were included when their use was relevant to expectation management, treatment evaluation, or communication during decision-making. Measurement of patient satisfaction or clinical outcomes alone was not regarded as evidence of improved SDM.

Eligible study designs included clinical or observational studies, randomized and nonrandomized controlled trials, prospective and retrospective comparative cohort studies, qualitative or mixed-methods studies, case series, technical validation studies with relevant decision-making outcomes, and original research articles regarding Large Language Model(LLM)-generated patient information which could influence patients’ decision-making outcomes. Additionally, studies were required to address aesthetic plastic surgery and cosmetic interventions contexts and to be published in the English language from 2011 through 2026. Systematic reviews and meta-analyses were examined to identify additional relevant references but were not included as main studies in the qualitative synthesis. In addition, the reference lists of the selected full-text articles and relevant systematic reviews or meta-analyses were scrutinized manually for additional relevant references.

Studies were excluded if they presented algorithm or tool developments without a decision support function, failed to report measurable outcomes, provided insufficient methodological information for quality assessment, focused mainly on surgical results without relevance to the process of decision-making, or addressed non-cosmetic interventions or surgeries outside the spectrum of aesthetic plastic surgery. Editorials, commentaries, conference abstracts, and study protocols, as well as articles written in other languages, were also excluded.

### 2.3. Data Collection

The retrieved citations were independently screened by two reviewers on the basis of title and abstract content, and in the next stage the selected articles underwent full-text evaluation. Any disagreements that arose during this process were resolved through discussion until consensus was achieved. Using the PubMed records as a basis for the initial search, any duplicate articles derived from Cochrane Library and Google Scholar were identified during abstract screening and excluded before full-text evaluation.

Data were extracted regarding the method of SDM, type of decision aid or interventional decision-support tool used, type of aesthetic intervention involved in the counseling process, main findings associated with SDM, as well as study information regarding authors, design, sample size, journal, and year of publication. Specifically for studies evaluating AI and large language model applications, data regarding evaluation approach, accuracy, readability, and clinical implementation were additionally recorded. Data collection was performed by one reviewer and subsequently verified by a second reviewer for accuracy and relevance. Any discrepancies were resolved through discussion and consensus.

### 2.4. Risk of Bias Assessment

The 38 included studies were appraised using appropriate risk of bias assessment tools according to study design, as the literature was characterized by significant heterogeneity. ROBINS-I tool was used to evaluate nonrandomized studies exploring the clinical effect of an intervention, while randomized studies were assessed using the Cochrane Risk of Bias 2 tool. Design-specific Joanna Briggs Institute critical appraisal tools were employed for eligible case series, cohort, cross-sectional, and qualitative studies, while the Prediction Model Risk of Bias Assessment Tool (PROBAST) was used to evaluate prediction model studies. Studies concerning LLM-generated patient information, imaging, and simulation accuracy were assessed in a descriptive manner in the context of standardization and reproducibility, question as well as reviewer selection, outcome recording, and generalizability (Supplementary Table S1) [5,8,9,10,11,12,13,14,15,16,17,18,19,20,21,22,23,24,25,26,27,28,29,30,31,32,33,34,35,36,37,38,39,40,41,42,43,44]. Due to the significant heterogeneity in study designs and outcomes, a statistical analysis of reporting bias was not undertaken.

### 2.5. Data Synthesis and Certainty Assessment

A stratified narrative synthesis was conducted considering the substantial variability in study designs, interventions, and measured outcomes. In this context, we distinguished (a) formal decision aids and SDM interventions, (b) patient-reported outcome measures, (c) 3D imaging and simulation applications, (d) predictive and risk-stratification models, and (e) LLM-derived patient information. The evidence was interpreted according to direct influence on SDM, patient experience and perceived evaluation, clinical outcomes, or technical performance indirectly affecting patient counselling and decision process. Comparative studies involving patient experience and satisfaction/decisional conflict measurement were bestowed with greater interpretive significance than uncontrolled cohorts, expert evaluations, or technical validation studies. The key findings of these studies, as well as their main characteristics and final recommendations, were also summarized (Supplementary Table S2) [5,8,9,10,11,12,13,14,15,16,17,18,19,20,21,22,23,24,25,26,27,28,29,30,31,32,33,34,35,36,37,38,39,40,41,42,43,44].

Meta-analysis or subgroup analysis was not conducted owing to the considerable heterogeneity of the available studies. No formal GRADE certainty rating was assigned considering the variability of evaluative questions employed and tool-specific domains encompassing the study assessments. Furthermore, technical validation studies and evaluation studies targeting LLM-generated patient information could not provide concrete evidence of impact on SDM or patient experience.

This systematic review was not registered in PROSPERO or another registry, and no formal protocol was prepared. Thus, eligibility criteria and analytic decisions cannot be independently verified.

## 3. Results

The initial literature query identified 5485 citations: 2239 from PubMed, 776 from Cochrane Library, and 2470 from Google Scholar, which were initially screened by title for topic relevance. At this stage, 5122 records were excluded (1981 from PubMed, 742 from Cochrane Library, 2399 from Google Scholar), while 363 articles were evaluated by abstract. Of 363 articles, 157 records derived from PubMed and 31 from Cochrane Library were excluded during abstract screening, while 67 duplicates from Cochrane Library and Google Scholar were also removed. Subsequently, a total of 108 articles underwent full-text evaluation to determine eligibility (101 from PubMed, 7 from Google Scholar). Studies were excluded primarily because they focused on surgical outcomes rather than the outcome of the SDM process, presented the development of decision aids, algorithms, and applications but lacked relevance to decision-making outcomes, or aimed at exploring the effect of SDM in disciplines outside the field of aesthetic plastic surgery and cosmetic interventions.

**Figure 1:**
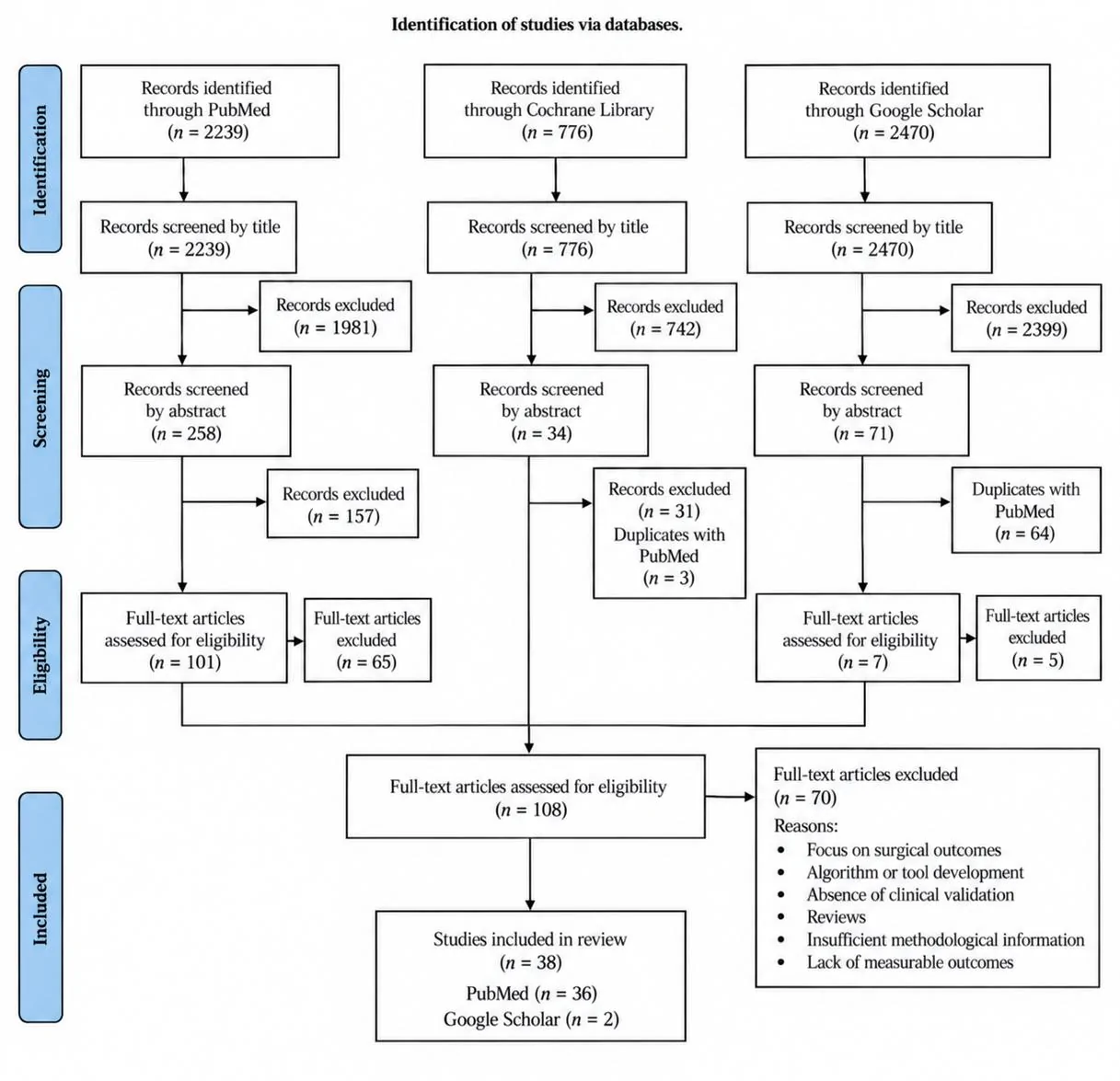
PRISMA flow diagram.

In total, 38 studies were included in the qualitative synthesis encompassing distinct evidence types. The final structure of selected articles encompassed randomized and nonrandomized studies, case series, prospective and retrospective observational studies, mixed-methods and qualitative studies, technical validation studies, and evaluations of LLM-derived information. The study selection process, including identification, screening, eligibility evaluation, and final study inclusion, is presented in the PRISMA flow diagram (Figure 1). Table 1 presents SDM strategies and interventional tools briefly, while Supplementary Table S2 provides an overview of the included studies and their key characteristics. Supplementary Table S1 presents the risk-of-bias assessment.

Direct decision-support evidence is concentrated in a small subgroup. Bian et al. developed a decision aid and piloted it with 20 patients, providing perceived decision-support findings, while Paraskeva et al. reported clarification of patient goals using the PEGASUS intervention in a sample of 17 participants [8,43]. Visualization and simulation studies mainly measured prediction agreement, accuracy, and perceived satisfaction without directly providing feedback on SDM outcomes. Basile et al. and Cui et al. evaluated predictive models, while Bukret et al. focused on risk stratification [25,41,42]. LLM studies, including Seth et al. and Yun et al., assessed the quality and accuracy of chatbot-generated responses to patients’ questions affecting patient knowledge [32,40]. However, both technical validation and AI evaluation studies failed to demonstrate concrete evidence regarding improved SDM or decisional conflict.

Overall, the authors evaluating SDM support tools reported an increase in perceived participation and improved patient understanding. Patients were capable of comprehending both their preferences as well as the available options and aligning their healthcare better with realistic expectations. However, the evidence supporting these outcomes was limited. Furthermore, the use of validated decision aids in routine aesthetic practice was rather restricted. The overwhelming majority of studies revolved around the use of AI technologies and LLM applications evaluating accuracy, usability, and patient preference, indicating potential utility and providing, thus, only indirect information which could be relevant to SDM.

Current literature presents considerable heterogeneity and variability across study designs, sample sizes, performance metrics, and methodology, while many studies place rather substantial emphasis on technical and algorithmic performance instead of clinical effectiveness. Most studies lacked sufficient datasets, external validation, consistent control groups, prospective clinical trials, and objective performance metrics, limiting the assessment of the models’ reliability and reproducibility. Supplementary Table S1 summarizes judgments on risk of bias assessment regarding each of the 38 studies according to design. Risk of bias was principally moderate to high across the available literature, and limitations were observed in terms of sample size, reviewer selection, blinding, follow-up, subjective scoring, overestimation of technical accuracy, and lack of clinical evaluation affecting the reliability and generalizability of reported findings. Although many studies presented adequate reporting of sample characteristics and clinical information, concerns regarding methodology occurred, particularly relevant to patient or question selection, outcome measurement, analysis of findings, and confounding factors. As a result of the absence of control groups, randomization, or standardized outcome measurements, the effectiveness of the described interventions could not be adequately verified. Technical evaluation studies were limited by inadequate external validation and missing data, while LLM studies were characterized by small expert panels, lack of patient participation, and subjective evaluations.

These findings suggest potential utility of these intervention tools in the context of SDM; however, lack of strong evidence restricts evaluation of overall effectiveness. Thus, the importance of conducting rigorous multicenter controlled validation studies is highlighted, analogous to the complexity of these models, and establishing standardized evaluation frameworks and long-term evaluation of clinical outcomes to establish their impact on SDM.

## 4. Discussion

### 4.1. Shared Decision-Making in Aesthetic Plastic Surgery

Shared decision-making reflects a notion that prioritizes respect for patient autonomy and a collaborative relationship between patient and surgeon aiming to align treatment decisions both with the current scientific facts and the patient’s personal principles and expectations [1]. Key elements encompassing the concept of SDM constitute: (1) active involvement of at least two participants, i.e., the physician and the patient; (2) mutual exchange of relevant information between the participants; (3) synergetic effort to reach unanimity regarding the most appropriate treatment option; and (4) agreement on the treatment plan to be carried out [2,45,46].

Aesthetic plastic surgery encompasses a domain of elective surgical and minimally invasive procedures, which aim to improve a person’s appearance and overall well-being. Hence, the patient’s desire to enhance his personal quality of life principally drives his requested treatments, which, indeed, constitute no medical necessity [2]. Failure to achieve the expected benefits may result in patient dissatisfaction, emotional distress, and potential legal action, even when the treatment is technically considered successful [8].

The balance between patients’ desires and realistic outcomes has become increasingly nuanced and fragile, bearing in mind the revolution brought on by a constantly expanding digitalization. The upcoming challenges regarding physician–patient communication and establishment of trust render the adoption of more refined approaches in counseling imperative [47]. As social media frequently promotes unattainable beauty standards, physicians play a pivotal role in helping patients develop realistic expectations that are consistent with achievable aesthetic results [48]. Owing to the considerable number of available options addressing a particular need, the implementation of SDM in the routine practice of aesthetic plastic surgery constitutes a valuable approach, which aims to engage patients in the process of decision-making in accordance with their values and preferences [46]. Besides, patient education and transparent communication contribute to addressing unrealistic expectations shaped by social media and identifying psychological vulnerabilities that may influence patients’ treatment decisions as well as the eventual outcome [48].

The goal of SDM is to direct the physician’s awareness toward apprehending the patients’ point of view in an efficient way beyond disclosure of information, to nurture patient understanding, and to effectively align the treatment option with their values. Patients initiated in the process of SDM, in addition to informed consent, express a higher level of satisfaction, limiting the risk of litigation and adhering to the treatment plan more effectively [2,49,50]. A systematic review by Niburski et al. verified the impact of SDM on surgical patients’ overall psychological well-being, accentuating a reduction in anxiety and decisional conflict concomitant with enrichment of satisfaction and trust [51]. Still, the implementation of SDM in aesthetic plastic surgery remains limited due to concerns of delay during consultation and workflow barriers, even though this claim is not supported by the literature, as well as misconceptions regarding informed consent as the sole necessary step toward efficient decision-making [2,4,46,52]. Research also shows that only a limited fraction of healthcare professionals sufficiently apprehend the concept of SDM, even though they support its purpose [8,46].

### 4.2. Decision Aids

The amalgamation of decision aids in the process of SDM has been associated with improvement in patients’ knowledge and perception of risks in some clinical settings, leading to enhanced decisional confidence [1]. Decision aids are evidence-based, educational tools that incorporate information about the situation concerning the patient, enumerate the available options, weigh their advantages and disadvantages as well as the potential risks, and present the anticipated outcomes associated with each option in a structured way [2,4]. Concurrently, the patients become capable of acknowledging their preferences and values and clarifying their goals and expectations [49]. The construction of decision aids is regulated by the guidelines provided by the International Patient Decision Aid Standards (IPDAS) and the delivery of content may take the form of printed booklets, digitalized brochures, interactive rating scales, visual agents and web-based applications [4,49].

Current literature involving reconstructive and oncologic populations supports the beneficial role of decision aids in SDM, portraying their efficacy in reducing decisional conflict and regret and improving patients’ knowledge [2,4,53,54]. Patients also reported significantly higher levels of confidence in the process of decision-making, while overtreatment was obviated [46,54]. The positive impact of decision aids has been reported in meta-analyses presenting higher levels of patient satisfaction, decreased levels of decisional conflict and regret, and increased involvement in the process of decision-making [55,56]. Zhong et al. reinforced the vital aspect of communication and patient education during preoperative counseling, reporting lower levels of decisional regret in patients expressing satisfaction with information received during the decision-making process [57].

In plastic surgery, the integration of decision aids in shared decision-making involved mainly the oncologic and reconstructive terrain. In the field of breast reconstruction, the implementation of decision aids in the process of SDM was correlated with lower levels of decisional conflict and regret combined with higher levels of patient contentment and education [4,58]. Patients exhibited greater understanding of their options and how these could be aligned to their preferences and values, alleviating, thus, the burden of psychological vulnerabilities that accompany their clinical state [59]. However, studies evaluating the role of decision aids in aesthetic plastic surgery remain limited, and further studies are needed to establish their impact on patient satisfaction and decisional conflict. Bian et al. conducted a pilot study evaluating the impact of a patient-centered decision aid on breast augmentation and breast reduction patients and reported improved communication and reduced decisional regret [43]. A retrospective study by Kolasinski et alpresented the utility of a decision aid developed for implant selection in the decision-making process in conjunction with 3D simulation [44].

### 4.3. Patient-Reported Outcome Measures

The use of validated quality-of-life metrics has been associated with improved treatment planning and outcomes. Patient-reported outcome measures (PROMs) such as BREAST-Q and FACE-Q constitute standardized and endorsed questionnaires that capture patients’ perception of their health status, functional and aesthetic outcomes, provide valuable insights into treatment efficiency and patient satisfaction, and could be leveraged to create a framework for more efficient communication in the counseling of subsequent patients [19,47,60,61,62,63]. PROMs do not constitute decision aids but may contribute to the decision-making process by taking into account the perceived experience of former patients and the results of comparing different options [64]. The impact of PROMs on patient satisfaction has been particularly evaluated in breast cancer patients facing the crossroads of breast reconstruction options without, however, demonstrating direct improvement in SDM, while sufficient research is still lacking in the field of aesthetic plastic surgery [57,65,66,67,68,69]. As our world is increasingly becoming digitalized, advances in technology have enabled the collection of patient-reported outcome data through electronic applications and platforms (e-PROMs) in an efficient and productive way [70].

### 4.4. Artificial Intelligence and Shared Decision-Making

Artificial intelligence (AI) encompasses computer systems and machines that possess the ability to process and interpret data, adapt to evolving conditions, learn, and make decisions in a manner that resembles human cognitive abilities [6,71]. These systems use the available knowledge absorbed from human research to provide assistance, evolving the whole spectrum of healthcare from counseling to procedural guidance, monitoring, documentation, research, education, and innovation [72]. In aesthetic plastic surgery, these technologies could be leveraged for patient education, preference elicitation through image analysis, and prediction of outcomes and treatment planning. The direct effect of AI-based technologies on SDM and patient satisfaction, though, remains to be further investigated.

#### 4.4.1. Machine Learning

Machine learning (ML) comprises a range of algorithms that learn from data and user input, identify patterns and extract valuable insights through contextual relationship recognition, develop predictive models, and estimate future outcomes [6,72]. In plastic surgery, ML algorithms are capable of analyzing and eliciting patterns from large-scale datasets, which may include medical imaging, clinical information, and patient demographic traits, while image interpretation enables the detection of anatomical landmarks, facial and body surface analysis, and simulation of surgical outcomes relevant to patient counseling [6,72,73].

#### 4.4.2. Predictive Models and Risk Stratification Tools

Predictive modeling encompasses a combination of statistical algorithms and machine learning methods that enable the exploration of available evidence, identification, and decryption of patterns to navigate the anticipation of specific outcomes [72,73]. In aesthetic plastic surgery, these technologies may provide the potential to improve risk prediction and surgical planning, while risk stratification tools may contribute to individualized guidance during consultation by generating patient-specific risk profiles that are aligned to their personal characteristics [73,74]. Their role in SDM depends on adequate validation and evidence that their use may result in better clinical outcomes. A study by Cui et al. in 361 participants reported high accuracy of the prediction model; however, a high risk of bias was concluded following PROBAST assessment [25]. Bukret et al. screened 3347 patients but reported outcomes for only a small sample, rendering the impact of AI risk stratification on complications difficult to evaluate [41].

#### 4.4.3. Large Language Models

The use of large language models (LLMs) has added a new aspect in patient education by providing information concerning patient inquiries on indications, risks, expected course, and outcomes, potentially enhancing patient understanding [7]. LLMs, such as ChatGPT (3.5 and 4, OpenAI, San Francisco, CA, USA), are designed in a way that enables them to deliver coherent and empathetic answers to patients’ concerns relevant to various aspects of plastic surgery in a conversational layout, a capability which may prove useful in aiding patient education, guidance, and individualization of care [24,75,76,77]. However, current available studies principally evaluate accuracy, quality and readability, lacking data on clinical efficacy, impact on SDM and patient satisfaction. Across the available studies, limited question sets, subjective expert scoring, and missing data in statistical analysis impede concrete conclusions.

#### 4.4.4. 3-Dimensional Imaging, Simulation and Virtual Reality

Conventional visualization tools, such as before-and-after photographs of other patients or external implant sizers, have traditionally been employed within the framework of consultation and shared decision-making to manage expectations [78]. The development of 3-dimensional (3D) imaging simulation emanated from the need to convey, on the one hand, complex clinical information into a more understandable format and alleviate, on the other hand, limitations emerging from the employment of conventional methods like implant sizers during the preoperative consultation [5,27,28,78]. Technologies, like augmented reality (AR), virtual reality (VR), facial recognition, and soft tissue simulation (STS), are increasingly being integrated into patient consultation with the aim of identifying anatomical landmarks following image analysis and providing visualization of their potential aesthetic outcomes, potentially substituting patients’ “before” and “after” photographs during counseling [9,20,79,80,81,82]. These technologies have been evaluated for simulation accuracy, preoperative planning, patient satisfaction, and preference elicitation, while studies demonstrate improved communication and patient understanding [14,16,17,20,83,84]. Patients are increasingly displaying a preference for consultations that incorporate simulation technology; however, its effect on shared-decision making requires further prospective evaluation and standardized measurement [19,20].

### 4.5. Challenges and Ethical Issues

The use of AI in aesthetic practice raises ethical and practical concerns regarding data privacy, transparency, cultural bias in aesthetic standards, and accountability for AI-derived recommendations and inaccuracies. These issues should be addressed in conjunction with assessment of these technologies’ clinical utility [6].

#### 4.5.1. Data Privacy

Aesthetic plastic surgery presents particular challenges in data privacy, as AI algorithms may access sensitive patient information including photographic content, personal health records and psychological evaluations. The acquisition and utilization of these data raise substantial ethical and privacy concerns unraveling beyond mere medical confidentiality [74]. Hence, the use of patient data to train and refine AI algorithms without explicit patient consent jeopardizes patient trust by risking identification as well as data exploitation by cybercriminals [85].

#### 4.5.2. Liability, Accountability, and Transparency

Researchers express their concern about issues of responsibility and liability, especially in situations where the clinical judgment of the physician does not accord with AI-generated recommendations [71]. Lack of human oversight, additionally, leaves users in the dark about the rationale that conveys the outcomes produced by AI, addressing multiple issues [6]. Absence of transparency concerning the origin of information generated raises concerns with regard to accuracy, reliability, and credibility [77,86,87,88]. In aesthetic practice, physicians are therefore required to interpret AI recommendations and inform their patients about limitations and risks.

#### 4.5.3. Biases and Homogenization

Important ethical concerns, especially those related to algorithmic bias affecting diverse demographic populations, arise owing to limited datasets that are employed in the training process of AI [7]. As a result of inequities encoded in the algorithms, underrepresented groups are not adequately evaluated, while suggested output may be regarded as misleading [7,73,74]. This concern is particularly pertinent to aesthetic applications, as beauty ideals are inherently influenced by cultural and ethnic diversities, while AI systems may favor specific characteristics based on their predefined dataset of beauty standards, leading to suggestions that are misaligned with patients’ goals pertaining to different cultural backgrounds [71]. As a result, applicability and generalizability of the AI algorithms across different populations may be limited [73,74].

#### 4.5.4. Inaccuracy

LLMs, like ChatGPT, have been reported to engender responses that may appear plausible but encompass inaccuracies, inconsistencies, and omission of important details [7,87,89,90,91]. The phenomenon of “AI hallucinations” pertains to a reported tendency of chatbots to generate well-structured, false information that appears logical and may endanger the safety of patients with inaccurate recommendations [85].

In aesthetic practice, inaccuracies have also been reported in AI visualization technologies, which may jeopardize patients’ trust and the framework of communication between patient and physician by forging unrealistic expectations that could lead to disappointment and liability issues [16,82,92,93,94]. As a result, such outputs require expert verification prior to use in patient counseling.

#### 4.5.5. Overtreatment

In terms of aesthetic evaluation, AI may analyze anatomical features; however, the concept of beauty and aesthesis may not be digitally replicated [95]. Research also shows that AI chatbots currently exhibit a tendency to recommend aesthetic procedures excessively, sounding the alarm for possible exacerbation of mental illness in vulnerable individuals, as the algorithms do not possess nuanced clinical judgment to interpret patients’ requests and comorbidities and rely on standardized training data [96]. As a result, idealized standards of beauty and aesthetics are often promoted, which may be homogenized, unrealistic, and constantly evolving, engendering unattainable expectations and patient dissatisfaction [97].

### 4.6. AI as a Complementary Tool in SDM

AI technologies may support certain aspects of patient counseling through advanced data analyses, predictive modeling, and sophisticated machine learning, showing also potential in improving shared-decision making and clinical outcomes [98]. However, evidence verifying their contribution in SDM remains deficient, suggesting in the meantime that AI should be handled as an ancillary component in the process of shared decision-making and not as a replacement for human judgment [6,20,85,98,99]. Human traits like compassion and empathy, which are irreplaceable in terms of establishing genuine communication between physician and patient, cannot be digitally simulated [71,100].

### 4.7. Risk of Bias and Methodological Limitations

The current review presents several limitations with regard to the significant heterogeneity in study design, type of strategy or tool investigated, method of outcome measurement and validation, rendering a further analysis of results challenging. The evidence included variable levels of intervention ranging from formal decision aids and PROMs to AI visualization technologies and LLM applications, which were interpreted according to patient satisfaction, direct influence on SDM, or accuracy and usability with relevance to patient support during counseling. Technical accuracy and utility were not, however, considered comparable to established improvement in SDM.

The methodological appraisal of the 38 included studies presents several limitations regarding the available evidence. Despite the potential utility of technologies in shared decision-making, the overall evidence is constrained by substantial heterogeneity and risk of bias. The majority of studies presented considerably limited sample sizes and numbers of evaluators, limited clinical validation, subjectivity in expert evaluation, missing data, and confounding factors, translating to a high risk of bias. The validity and generalizability of the findings remain constrained due to a lack of randomized controlled trials with adequate samples, absence of control groups, reporting of subjective assessments, and influence of confounding factors. Studies evaluating AI applications and LLM-generated information are characterized by limited expert panels and absence of patient participation, and although technical feasibility and accuracy is reported correspondent findings should not be interpreted as evidence of clinical effectiveness and improved SDM. Addressing these methodological limitations, as well as evaluating the clinical performance of the described interventions, is essential to explore their impact on patient care and SDM.

Furthermore, eligibility was restricted to articles written in the English language, adding language bias to the review. With regard to search strategy, the use of the Google Scholar database introduces a reproducibility limitation given that its indexing criteria may change over time.

The review was not prospectively registered, and no formal protocol was prepared. As further meta-analysis could not be conducted and GRADE certainty was not formally evaluated by outcome, the strength of conclusions is rather limited. The overall effectiveness of SDM strategies and decision tools in the field of aesthetic plastic surgery and cosmetic interventions could not be efficiently confirmed, and evidence deriving from oncologic and reconstructive samples cannot be assumed transferable to aesthetic populations.

## 5. Conclusions

Our review accentuates a lingering gap in the implementation of SDM and standardized decision aids in the field of aesthetic plastic surgery. Current evidence cannot establish a connection between integrating modern technologies such as 3D imaging, virtual reality, predictive analytics, and large language models with improvement in patient counseling, shared decision-making, or patient satisfaction [7]. Addressing methodological and ethical concerns and conducting prospective multicenter studies with external validation are necessary steps prior to considering these technologies established tools in SDM. At the same time, physicians must remain vigilant to the risks of over-treatment and developing unrealistic expectations, prioritizing patients’ well-being and embracing evolving technologies with a critical mind and allegiance to ethical standards [97].

### Future Perspectives

Future studies should assess the implementation of standardized decision aids and decision-support interventions across the full range of aesthetic plastic surgery and their effect on patient–clinician communication, patient satisfaction and decisional regret, as well as congruence of decisions with patients’ values and preferences. Additionally, forthcoming research should invest in properly designed prospective and randomized controlled trials, standardized protocols, and multicenter collaborative research to determine the effectiveness of decision-support interventions and AI technologies, including their impact on shared decision-making, eventual outcomes, and cost-effectiveness, while ethical concerns accompanying their implementation should also be properly addressed.

## Figures and Tables

**Table 1:** SDM strategies and tools.

Category	Examples	Role in SDM ^1^
Educational Aids	Brochures, videos, websites	Enhance patient knowledge, refine focus of consultation
Decision Aids	Worksheets, interactive rating scales, risk visualization graphics, web-based applications	Increase comprehension of available treatments, risks, and benefits, clarify expectations
PROMs ^2^	BREAST-Q, BODY-Q, FACE-Q	Capture patients’ values, measure satisfaction with outcomes
AI ^3^ visualization tools	3D ^4^ imaging, AR ^5^, VR ^6^	Convey evidence in a comprehensible manner, potential risk communication and decision support
Machine Learning, Predictive Models	Simulation, risk stratification tools	Potential personalized decision support, risk prediction, individual profile development
Large Language Models	Chatbots	Increase patients’ knowledge, offer individualized guidance

^1^ SDM: Shared Decision-Making, ^2^ PROMs: Patient-Reported Outcome Measures, ^3^ AI: Artificial Intelligence, ^4^ 3D: 3-Dimensional, ^5^ AR: Augmented Reality, ^6^ VR: Virtual Reality.

## Data Availability

Additional data are provided within the Supplementary Materials.

## Supplementary Materials

The following supporting information can be downloaded at: https://www.mdpi.com/article/10.3390/healthcare14213672/s1, Table S1: Risk of bias assessment and methodological appraisal of included studies; Table S2: Overview of decision aids (DAs), methods of SDM and studies’ key characteristics.

## Abbreviations

The following abbreviations are used in this manuscript:

3D	3-Dimensional
AI	Artificial intelligence
AR	Augmented reality
DA	Decision aid
LLM	Large language model
ML	Machine learning
PROM	Patient-reported outcome measure
SDM	Shared decision-making
STS	Soft tissue simulation
VR	Virtual reality
