# Supplementary material for: Shared Decision-Making in Cosmetic Procedures and Aesthetic Plastic Surgery: A Systematic Review of Decision Aids, Clinical Benefits, Ethical Challenges, and Future Perspectives
Source: Healthcare (Basel). 2026 Oct 29;14(21):3672. doi: 10.3390/healthcare14213672 (PMC13763302; doi:10.3390/healthcare14213672)
Supplement: Supplementary file 1 [file healthcare-14-03672-s001.zip › revised supplementary_material_TableS1.pdf]

Supplemental Table S1. Risk of bias assessment and methodological appraisal of included studies

Author	Methodology	Risk of Bias Tool	Domain-level assessment	Key limitations
Paraskeva et al (2017), [8]	Mixed methods study	Joanna Briggs Institute critical appraisal tool for qualitative research	Congruity of philosophy: Unclear, Methodology: Yes, Data collection: Yes, Data representation: Yes, Interpretation: Yes, Cultural positioning: Unclear, Influence on researcher: Unclear, Participant voice: Yes, Ethical approval: Yes, Conclusions grounding: Yes	Mixed-methods PEGASUS feasibility, 17 women and 3 providers; no intervention comparator.
Lava et al (2025), [9]	Single institution prospective case series	Joanna Briggs Institute critical appraisal tool for case series	Inclusion criteria: Yes, Standardized measurement: Yes, Valid identification: Unclear, Consecutive inclusion: Unclear, Complete inclusion: Unclear, Demographics reporting: Yes, Clinical information: Yes, Outcomes/Follow-up: Unclear, Site demographics: Yes, Statistical analysis: No	7 operated patients, small uncontrolled simulation experience, no control group, reporting inconsistencies, lack of validation
Lorange et al (2026), [10]	Prospective cohort study	Technical validation appraisal	No validated RoB assessment applicable, Sample: Concern, Exclusion of complications: Concern, Standardized measurement: Yes, Assessor independence: Yes, Missing data: Concern, Criteria: Yes, Statistical analysis: Yes, External validation: Concern, Direct SDM benefit: Unclear	78 technical volume simulations, agreement endpoint not patient SDM, single center, exclusion of revisions, limited generalizability
de Runz et al (2018), [11]	Single institution case series	Joanna Briggs Institute critical appraisal tool for case series	Inclusion criteria: Yes, Standardized measurement: Yes, Valid identification methods: Yes, Consecutive participant inclusion: Unclear, Complete participant inclusion: Unclear, Participant demographics reporting: Yes, Clinical information reporting: Yes, Outcomes reporting: Yes, Site reporting: Yes, Statistical analysis: Unclear	38-patient uncontrolled simulation attitudes, subjective outcomes
Hutchinson et al (2021), [12]	Retrospective controlled cohort study	Joanna Briggs Institute critical appraisal tool for quasi-experimental studies	Temporal clarity: Yes, Participant similarity: No, Treatment similarity: Unclear, Control group: Yes, Multiple measurements: No, Complete follow-up: No, Outcome measurement consistency: Yes, Outcome measurement reliability: Unclear, Statistical analysis: No	Small sample, limited response rate, follow-up inconsistences, unclear SDM outcome
Hammond et al (2022), [13]	Single institution cohort study	Joanna Briggs Institute critical appraisal tool for case series	Inclusion criteria: Yes, Standardized measurement: Yes, Valid identification methods: Unclear, Consecutive participant inclusion: Unclear, Complete participant inclusion: No, Participant demographics reporting: Yes, Clinical information reporting: Yes, Outcomes reporting: Yes, Site reporting: Unclear, Statistical analysis: Unclear	40 consecutive patients, sizing methods without untreated comparator, single surgeon, subjective questionnaire, missing data
Yamamichi et al (2025), [14]	Retrospective cohort study	Technical validation appraisal	No validated RoB assessment applicable, Sample: Concern, Patient-reported outcomes: Yes, Standardized measurement:	Retrospective 38-patient simulation/clinical comparison,

			Yes, Assessor independence: Partial, Missing data: Concern, Subjective ratings: Unclear, Statistical analysis: Yes, Follow-up: Concern, Confounding: Concern, Direct SDM benefit: Unclear	single center, no control group, subjective scoring, variable follow-up
Persing et al (2018), [15]	Retrospective validation study	Technical validation appraisal	No validated RoB assessment applicable, Sample: Concern, Objective: Yes, Standardized measurement: Partially, Assessor independence: Yes, Criteria: Yes, Missing data: Unclear, Subjective ratings: Concern, Statistical analysis: Partially, Direct SDM benefit: Unclear	Retrospective 40-patient simulation agreement, assessor-dependent, single surgeon, participant selection, unreliable aesthetic scale
Demir et al (2024), [16]	Retrospective study	Technical validation appraisal	No validated RoB assessment applicable, Sample: Concern, Objective: Yes, Standardized measurement: Yes, Assessor independence: Unclear, Criteria: Partially, Operator influence: Concern, Statistical analysis: Yes, Direct SDM benefit: Unclear	Measurement comparison, technical planning accuracy, single surgeon, specifically selected patients, limited blinding
Roostaeian et al (2014), [17]	Single-surgeon case series	Technical validation appraisal	No validated RoB assessment applicable, Sample: Partially, Objective: Yes, Standardized measurement: Yes, Assessor independence: Partially, Criteria: Yes, Missing data: Concern, Statistical analysis: Unclear, Direct SDM benefit: Unclear	20 technical simulations, single surgeon, selection limitations, no clinical SDM contrast.
Bansal et al (2026), [18]	Single institution case series	Joanna Briggs Institute critical appraisal tool for case series	Inclusion criteria: Unclear, Standardized measurement: Yes, Valid identification methods: Yes, Consecutive participant inclusion: Unclear, Complete participant inclusion: Unclear, Participant demographics reporting: Yes, Clinical information reporting: Yes, Outcomes reporting: Yes, Site reporting: Unclear, Statistical analysis: Yes	3 patient case series, single surgeon, unclear inclusion criteria, measurement concerns
Overschmidt et al (2018), [19]	Prospective study with randomized and nonrandomized arms	Cochrane Risk of Bias 2 tool, ROBINS-I	D1 randomization: Some concerns, D2 deviations: Some concerns, D3 missing data: Some concerns, D4 outcome measurement: High risk, D5 selection of reported result: Some concerns.<br>D1 confounding: Serious, D2 participant selection: Moderate, D3 classification of interventions: Moderate, D4 deviations: NI, D5 missing data: NI, D6 outcome measurement: Moderate, D7 selection of reported result: NI	RCT n=23 (13 control, 10 simulation) and nonrandomized n=77, selection criteria, limited generalizability
Donfrancesco et al (2013), [20]	Single-center prospective and retrospective study	Joanna Briggs Institute critical appraisal tool for case series	Inclusion criteria: Yes, Standardized measurement: Yes, Valid identification methods: Yes, Consecutive participant inclusion: Yes, Complete participant inclusion: Unclear, Participant demographics reporting: Yes, Clinical information reporting: Yes, Outcomes reporting: Yes, Site	150 prospective augmentation patients and retrospective subset, no control group, subjective patient ratings, single center

			reporting: Yes, Statistical analysis: Unclear	
Van Waeyenberge et al (2026), [21]	Retrospective cross-sectional study	Joanna Briggs Institute critical appraisal tool for cross-sectional studies series	Inclusion criteria: Yes, Subjects and Setting: Yes, Exposure measurement: Yes, Condition criteria: Unclear, Confounding factors: No, Confounding strategies: No, Outcome measurement: No, Statistical analysis: Yes	Retrospective cross-sectional 126 simulation respondents, nonvalidated Likert outcome, single surgeon, no control group, limited generalizability
Lekakis et al (2019), [22]	Single-center study	Joanna Briggs Institute critical appraisal tool for cross-sectional studies	Inclusion criteria: Yes, Subjects and Setting: Unclear, Exposure measurement: Yes, Condition criteria: Unclear, Confounding factors: No, Confounding strategies: No, Outcome measurement: Unclear, Statistical analysis: Unclear	172 sequential 2D then 3D morphing participants, nonrandomized order, subjective outcomes, single center
Bhat et al (2022), [23]	Pilot study	Joanna Briggs Institute critical appraisal tool for cross-sectional studies	Inclusion criteria: No, Subjects and Setting: Unclear, Exposure measurement: Unclear, Condition criteria: NA, Confounding factors: Yes, Confounding strategies: No, Outcome measurement: Unclear, Statistical analysis: Yes	26 practitioner survey respondents, no patient outcomes, measurement concerns, participant selection criteria, subjective scoring
Marcaccini et al (2025), [24]	Prospective observational study	AI descriptive appraisal	No validated RoB assessment applicable, Question selection: Concern, Sample: Concern, Replicability: Partially, Reference standard: Concern, Rater characteristics reporting: Partially, Blinding: Unclear, Criteria: Concern, Analysis: Concern, SDM benefit: Unclear	Subjective ratings, no patient participation, limited evaluator panel, limited reproducibility no standardization in evaluation, limited generalizability, no blinding
Cui et al (2025), [25]	Multi-center observational study	Prediction Model Risk Of Bias Assessment tool (PROBAST)	Data source: Yes, Participants: Probably No, Defined predictors: Probably Yes, Predictors without knowledge of outcome: NI, Predictors available at intended time: No, Outcome definition: Probably Yes, Outcome measurement: Probably Yes, Blinding: NI, Outcome determination similarity: Probably Yes, Interval between prediction and outcome: Probably No, Sample size: NI, All participants included in analysis: Probably No, Missing data: Probably No, Multivariable analysis: Probably Yes, Performance measures evaluation: Probably No, Overfitting addressed: Probably No, Predictors correspondence to final model: NI	High concern participants, limited external validation, subjective outcomes
Barbon et al (2025), [26]	Retrospective single-center comparative study	ROBINS-I	D1 Confounding: Serious concern, D2 Participant selection: Serious concern, D3 Classification of interventions: Low concern, D4 Deviations: NI, D5 Missing Data: NI, D6 Outcome measurement: Moderate concern, D7 Selection of reported result: NI	Small sample, participant selection, lack of validated satisfaction scale, limited statistical analysis, limited outcome measurement

La Padula et al (2022), [27]	Prospective observational study	Joanna Briggs Institute critical appraisal tool for case series	Inclusion criteria: Yes, Standardized measurement: Unclear, Valid identification: Unclear, Consecutive inclusion: Unclear, Complete inclusion: Unclear, Demographics reporting: Yes, Clinical information: Yes, Outcomes/Follow-up: Yes, Site demographics: Unclear, Statistical analysis: Unclear	Small sample, selected population, lack of comparator, single surgeon, reporting concerns, incomplete follow-up
Pruksapong et al (2026), [28]	Prospective cohort study and randomized controlled trial	Cochrane Risk of Bias 2 tool	D1 randomization: Some concerns, D2 deviations: Some concerns, D3 missing data: Some concerns, D4 outcome measurement: High risk, D5 selection of reported result: Some concerns	Reporting inconsistencies, subjective evaluation, high risk of bias
Ersan et al (2026), [5]	Retrospective observational study	Joanna Briggs Institute critical appraisal tool for case series	Inclusion criteria: Yes, Standardized measurement: Unclear, Valid identification: Yes, Consecutive inclusion: Unclear, Complete inclusion: Unclear, Demographics reporting: Yes, Clinical information: Yes, Outcomes/Follow-up: Yes, Site demographics: Unclear, Statistical analysis: Unclear	No comparator, differences in sample characteristics, missing reporting data
Işıktekin et al (2026), [29]	Cross-sectional comparative study	AI descriptive appraisal	No validated RoB assessment applicable, Question selection: low, reproducibility: low, Reference standard: some concerns, Blinding: low, Analysis: some concerns, Generalizability: high	Subjective evaluation, limited generalizability, reporting limitations
Shiraishi et al (2024), [30]	Observational study	AI descriptive appraisal	No validated RoB assessment applicable, Question selection: low, reproducibility: some concerns, Reference standard: some concerns, Blinding: some concerns, Analysis: some concerns, Generalizability: high	Limited expert panel and evaluators, absent patient validation, limited question selection, unclear blinding, external validation concerns
Copeland-Halperin et al (2023), [31]	Exploratory study	AI descriptive appraisal	No validated RoB assessment applicable, Question selection: low, reproducibility: low, Reference standard: some concerns, Blinding: some concerns, Analysis: some concerns, Generalizability: high	Limited expert panel, lack of statistical analysis, outcome measurement concerns, unclear blinding
Seth et al (2023), [32]	Qualitative study	AI descriptive appraisal	No validated RoB assessment applicable, Question selection: some concerns, reproducibility: some concerns, Reference standard: some concerns, Blinding: some concerns, Analysis: low, Generalizability: high	Evaluator selection, limited expert panel, single center, no patient participation
Zhang et al (2025), [33]	Comparative study	AI descriptive appraisal	No validated RoB assessment applicable, Question selection: low, reproducibility: some concerns, Reference standard: some concerns, Blinding: low, Analysis: some concerns, Generalizability: high	Limited expert panel, limited question selection, subjective evaluation, no patient participation, limited statistical analysis reporting

Radulesco et al (2025), [34]	Comparative observational study	AI descriptive appraisal	No validated RoB assessment applicable, Question selection: some concerns, reproducibility: some concerns, Reference standard: some concerns, Blinding: some concerns, Analysis: some concerns, Generalizability: high	Limited expert panel, lack of validated evaluation system, single center, limited generalizability
Shao et al (2025), [35]	Comparative study	AI descriptive appraisal	No validated RoB assessment applicable, Question selection: low, reproducibility: some concerns, Reference standard: low, Blinding: low, Analysis: some concerns, Generalizability: high	Limited expert panel, no patient participation
Brito et al (2024), [36]	Observational study	AI descriptive appraisal	No validated RoB assessment applicable, Question selection: some concerns, reproducibility: some concerns, Reference standard: some concerns, Blinding: some concerns, Analysis: some concerns, Generalizability: high	Limited evaluators, no blinding, outcome measurement concerns, limited question selection, no patient participation
Bastaninejad et al (2026), [37]	Comparative study	AI descriptive appraisal	No validated RoB assessment applicable, Question selection: some concerns, reproducibility: some concerns, Reference standard: some concerns, Blinding: some concerns, Analysis: some concerns, Generalizability: high	Limited evaluators, selection of evaluators, limited question selection, subjective evaluation, no patient participation
Rivera et al (2026), [38]	Comparative study	AI descriptive appraisal	No validated RoB assessment applicable, Question selection: low, reproducibility: low, Reference standard: low, Blinding: low, Analysis: some concerns, Generalizability: high	Limited expert panel, no blinding, no patient participation, selection of evaluators
Abi-Rafeh et al (2024), [39]	Observational study	AI descriptive appraisal	No validated RoB assessment applicable, Question selection: some concerns, reproducibility: some concerns, Reference standard: some concerns, Blinding: some concerns, Analysis: some concerns, Generalizability: high	Limited expert panel, no patient participation, subjective evaluation
Yun et al (2023), [40]	Qualitative study	AI descriptive appraisal	No validated RoB assessment applicable, Question selection: some concerns, reproducibility: some concerns, Reference standard: low, Blinding: some concerns, Analysis: some concerns, Generalizability: high	Limited evaluators, limited representativeness, subjective assessment, no patient participation, confounding
Bukret et al (2025), [41]	Prospective observational study	Joanna Briggs Institute critical appraisal tool for cohort studies	Group similarity: Unclear, Exposure measurement similarity: NA, Exposure measurement Validity: Unclear, Confounding factors: Yes, Confounding strategies: Unclear, Baseline outcome-freedom: Unclear, Outcome measurement: Unclear, Follow-up duration: Yes, Follow-up completeness: Unclear, Strategies for incomplete follow-up: Unclear, Statistical analysis: Unclear	Patient selection, variable follow-up, small sample, no control group, substantial confounding, high risk of bias in analysis domain and reviewer judgment
Basile et al (2024), [42]	Retrospective validation study	Prediction Model Risk Of Bias Assessment tool (PROBAST)	Data source: Probably Yes, Inclusion/Exclusion criteria: Unclear, Participants: Unclear, Defined predictors: Probably Yes, Predictors without knowledge of outcome: NI,	Subjective measurement, lack of external validation

			Predictors available at intended time: Probably Yes, Outcome definition: Unclear, Outcome measurement: Unclear, Blinding: NI, Outcome determination similarity: Probably Yes, Interval between prediction and outcome: Probably Yes, Sample size: Unclear, All participants included in analysis: Unclear, Missing data: NI, Multivariable analysis: Unclear, Performance measures evaluation: Probably No, Overfitting addressed: Unclear, Predictors correspondence to final model: Unclear	
Bian et al (2025), [43]	Pilot study	Joanna Briggs Institute critical appraisal tool for case series	Inclusion criteria: Yes, Standardized measurement: Unclear, Valid identification: Unclear, Consecutive inclusion: Unclear, Complete inclusion: Unclear, Demographics reporting: Yes, Clinical information: Yes, Outcomes/Follow-up: Unclear, Site demographics: Unclear, Statistical analysis: Unclear	Small sample, no control group, single center, reporting outcome concerns, subjective evaluation
Kolasiński et al (2025), [44]	Single-center retrospective cohort study	Joanna Briggs Institute critical appraisal tool for case series	Inclusion criteria: Yes, Standardized measurement: Yes, Valid identification: Unclear, Consecutive inclusion: Unclear, Complete inclusion: Unclear, Demographics reporting: Yes, Clinical information: Yes, Outcomes/Follow-up: Yes, Site demographics: Yes, Statistical analysis: Yes	No comparator, participant selection, missing data

RoB: Risk of bias, NA: Not applicable, NI: No information
