# Supplementary material for: Shared Decision-Making in Cosmetic Procedures and Aesthetic Plastic Surgery: A Systematic Review of Decision Aids, Clinical Benefits, Ethical Challenges, and Future Perspectives
Source: Healthcare (Basel). 2026 Oct 29;14(21):3672. doi: 10.3390/healthcare14213672 (PMC13763302; doi:10.3390/healthcare14213672)
Supplement: Supplementary file 1 [file healthcare-14-03672-s001.zip › revised supplementary_material_TableS2.pdf]

Supplemental Table S2. Overview of decision aids (DAs), methods of SDM and studies' key characteristics.

Author	Title	Published in/Country	SDM strategy/ Decision aid type	Type of procedure	Methodology	Key Findings	Recommendations
Paraskeva et al (2017), [8]	Facilitating shared decision-making with breast augmentation patients: Acceptability of the PEGASUS intervention	Journal of Plastic, Reconstructive & Aesthetic Surgery - UK	Patients' Expectations and Goals: Assisting Shared Understanding of Surgery (PEGASUS) Intervention: The patients list their goals regarding the surgery on a sheet and rate the importance of each (from 0 to 10)	Breast augmentation	Mixed methods study	17 female participants. Participants supported the relevance of the tool. Clarification of their goals and identification of realistic expectations.	Future research could explore the use of PEGASUS with patients seeking other aesthetic procedures
Lava et al (2025), [9]	The Role of Decision Aids and Soft Tissue Simulations in Breast Reduction Surgery: A Pilot Study	Plastic and Reconstructive Surgery Global Open - USA	3-D modeling and Soft Tissue Simulation (STS) (MirrorMe3D) plus BREAST-Q	Breast reduction	Single institution prospective case series	7 female participants. STS provided a visual representation supporting the process of decision-making.	Prospective comparative study to assess the impact of STS on patient satisfaction, decisional conflict, and postoperative regret.
Lorange et al (2026), [10]	3-Dimensional Simulation for Breast Augmentation: Does the Software Actually Work?	Plastic and Reconstructive Surgery - Canada	3-D imaging (Vectra XT 3D imaging system)	Breast augmentation	Prospective cohort study	78 patients. Vectra can accurately predict postoperative breast volumes after breast augmentation within a 10% margin of error. Accuracy decreased in larger breasts.	Refine AI systems in breast surgery and improve the accuracy of predictive models while further investigating their influence on patient satisfaction.
de Runz et al (2018), [11]	Three-dimensional imaging, an important factor of decision in breast augmentation	Annales de Chirurgie Plastique Esthétique - France	3-D imaging (Crisalix) Vs fitting of implants in a bra	Breast augmentation	Single institution case series	38 women. 93% of patients agreed that the 3-D imaging system helped in implant decision. 50% prefered 3-D simulation over classic fitting of implants in a bra.	3-D simulation is useful in outcome visualization but should be used as a complement in decision-making alongside classic

							method of trying with different implants in a bra.
Hutchinson et al (2021), [12]	A Pilot Study Evaluation of 3-Dimensional Imaging in Cosmetic Breast Augmentation: Results of a Single Surgeon 3.5-Year Retrospective Study Using the BREAST-Q Questionnaire	Aesthetic Surgery Journal Open Forum - USA	3-D imaging (VECTRA Volumetric 3D Surface Imaging System) plus BREAST-Q postoperatively	Breast augmentation	Retrospective cohort study	54 participants, 26 patients in the study group and 28 patients in the control group. Improved patient satisfaction with outcome and surgeon as well as physical well-being.	Future studies involving a larger sample size to determine a significant statistical significance.
Hammond et al (2022), [13]	Use of Three-Dimensional Imaging to Assess the Effectiveness of Volume as a Critical Variable in Breast Implant Selection	Plastic and Reconstructive Surgery - USA	3-D imaging (Crisalix Virtual Aesthetics), "before-and-after" photographs of previous patients, in-depth verbal discussion, external silicone sizers in a bra	Breast augmentation	Single institution cohort study	40 participants. 3-D simulation with virtual reality tool and external sizers were the prefered methods that facilitated the choice of implant. The majority of patients considered the addition of 3-D simulation and virtual reality during shared decision making as very helpful and significant (62% and 88% respectively).	The use of 3-D imaging and virtual reality facilitates communication during implant selection. Special emphasis should be given to the factor of volume in order to achieve better levels of patient satisfaction.
Yamamichi et al (2025), [14]	Three-Dimensional Simulation Accuracy and Patient Satisfaction With Rhinoplasty	Aesthetic Surgery Journal Open Forum - Japan	3-D imaging (Crisalix) plus FACE-Q	Rhinoplasty	Retrospective study	38 patients. Researchers reported high predictive accuracy significantly associated with greater patient satisfaction.	Future evolution of 3-D simulation tools will provide more precise and personalized outcomes.
Persing et al (2018), [15]	Three-Dimensional Imaging in	Aesthetic Surgery Journal	3-D imaging (Vectra 3D imaging system)	Rhinoplasty	Retrospective study	40 patients, 34 female and 6 male patients. The actual	Conduction of a study concerning patient

	Rhinoplasty: A Comparison of the Simulated versus Actual Result	- USA				postoperative outcome was preferred significantly more than the simulated result.	satisfaction for the simulated versus actual postoperative result.
Demir et al (2024), [16]	Optimizing Implant Width Selection in Breast Augmentation: Insights From On-Patient Landmark Positioning in 3-Dimensional Breast Simulation	Aesthetic Surgery Journal - Turkey	3-D imaging (Vectra XT 3D imaging system)	Breast augmentation	Retrospective study	69 female participants. 3-D simulation may lead to inaccuracies in the evaluation of breast dimensions owing to automatic detection of landmarks.	Clinical judgement is irreplaceable and physicians should manually position the anatomic landmarks on the patient prior to simulation.
Roostaeian et al (2014), [17]	Three-Dimensional Imaging for Breast Augmentation: Is This Technology Providing Accurate Simulations?	Aesthetic Surgery Journal - USA	3-D imaging (Vectra M3 3D imaging system)	Breast augmentation	Single-surgeon case series	20 patients. The imaging system demonstrated high predictive accuracy (90% for breast volume and 98,4% for contour)	Larger-scale studies are necessary to detect discrepancies in prediction and further refinement of the imaging systems is recommended.
Bansal et al (2026), [18]	Augmentation-First Approach for Breast Asymmetry Correction Using Crisalix 3D Imaging	Annals of Plastic Surgery - India	3-D imaging (Crisalix) plus external sizers	Breast augmentation for breast asymmetry	Single institution case series	3 patients. High patient satisfaction was noted (average 4,7/5)	Future studies should incorporate advanced imaging and preoperative tools to further improve patient satisfaction and surgical accuracy.
Overschmidt et al (2018), [19]	A Prospective Evaluation of Three-Dimensional Image Simulation: Patient-Reported Outcomes and Mammometrics in Primary Breast Augmentation	Plastic and Reconstructive Surgery - USA	3-D imaging (Vectra Sculptor) plus BREAST-Q	Breast augmentation	Prospective study with randomized and nonrandomized arms	100 patients, 23 constituted the randomized arm and 77 the nonrandomized arm. The authors found no statistical significance in patient satisfaction and patient-reported outcomes between nonsimulated and simulated groups, randomized or not. The communication between	Conducting a a prospective, randomized, controlled trial in an aesthetic patient population with longer follow-up period.

						patient and surgeon cannot be digitally replicated.	
Donfrancesco et al (2013), [20]	Three-Dimensional Simulated Images in Breast Augmentation Surgery: An Investigation of Patients' Satisfaction and the Correlation between Prediction and Actual Outcome	Plastic and Reconstructive Surgery - Sweden	3D imaging (Vectra 3D imaging system and Precision Light Software)	Breast augmentation	Single-center prospective and retrospective study	150 patients took part in the prospective study, 52 patients were included in the retrospective study. The majority of patients (86%) reported high accuracy of simulation as opposed to actual result and comparable percentages of high satisfaction with the simulation and decision-making were reported.	Further research including multi-center randomized controlled trials, systematic reviews and meta-analyses. Technology should be used as a complementary tool to a surgeon's judgment in shared decision-making.
Van Waeyenberge et al (2026), [21]	3D simulation in primary breast augmentation: a real-world study of patient-reported outcomes in early independent practice	Annals of Breast Surgery - Belgium	3D imaging (Vectra 3D Imaging)	Breast Augmentation	Retrospective cross-sectional study	126 female participants. The majority of participants considered the 3D simulation as reliable. While 92,1% of the patients would recommend 3D simulation during counseling.	3D simulation is a valuable tool for shared decision-making and expectation management. Standardization of 3D simulation protocols is recommended for more consistent results.
Lekakis et al (2019), [22]	Three-dimensional Morphing and Its Added Value in the Rhinoplasty Consult	Plastic and Reconstructive Surgery Global Open - Belgium	2-D simulation (Photoshop CS3 Extended Version 10) plus 3-D imaging (Vectra H1 handheld imaging system)	Rhinoplasty	Single-center study	172 participants. The majority of patients valued 3-D simulation over 2-D morphing during their preoperative counseling. 3-D simulation constitutes a useful method during preoperative evaluation and decision-making.	Patients should be thoroughly informed during consultation so that the development of unrealistic expectations may be avoided.
Bhat et al (2022), [23]	Visual Preoperative Risk Depiction Tools for Shared Decision-making: A Pilot Study from the Surgeon's	Plastic and Reconstructive Surgery Global Open - Australia	Risk visualization graphics	Reduction mammoplasty	Pilot study	26 participants. Risk visualization graphics support shared decision-making by effectively presenting relevant risks alongside benefits.	Different surgical centers should conduct a randomized trial to illuminate the utility of these tools.

Marcaccini et al (2025), [24]	Perspective Enhancing patient education in breast surgery: artificial intelligence-powered guidance for mastopexy, augmentation, reduction, and reconstruction	Translational Breast Cancer Research - Italy and Australia	Large Language Model (ChatGPT)	Mastopexy, Breast augmentation, Breast reduction, and Breast reconstruction	Prospective observational study	The responses were evaluated by 3 plastic surgeons, 1 nurse and 2 patients. ChatGPT enhanced patient education abd communication by providing simplified and clear responses to patients' concerns. The use of ChatGPT necessitates human oversight and serves as complementary tool in the shared-decision making process.	Further refinements regarding continuous training feedback as well as enhanced personalization are required, while research in real-world clinical scenarios and trials should be conducted.
Cui et al (2025), [25]	Prognostic assessment and intelligent prediction system for breast reduction surgery using improved swarm intelligence optimization	Frontiers in Medicine - China	Automated Machine Learning (Intelligent prediction system with Improved Secretary Bird Optimization Algorithm)	Breast reduction	Multi-center study	361 participants comprised the training data set. The proposed prediction system could accurately predict postoperative outcomes and quality of life enabling optimization of treatment planning.	Future research should prioritize external validation and the development of models to enhance the accuracy and clinical applicability of these systems.
Barbon et al (2025), [26]	Pushing Boundaries in Aesthetic Breast Surgery: Precise Surgical Planning With Online Consultation and 3-dimensional Simulation	Plastic and Reconstructive Surgery Global Open - Switzerland	Telemedicine and 3-D Simulation (Arbrea Breast Software)	Mastopexy, Breast augmentation and Breast reduction	Retrospective single-center study	15 participants in the online consultation with 3-D imaging. The postoperative outcomes were accurate and conversion rate was unaffected. Patients reported satisfaction and geographic barriers were negated.	Future prospective studies involving larger patient cohorts and longer follow-up periods are necessary to validate these findings and establish their long-term clinical applicability.
La Padula et al (2022), [27]	Assessment of Patient Satisfaction Using a New Augmented Reality Simulation Software for Breast	Journal of Clinical Medicine - Italy	3-D Simulation (Arbrea Breast Software) plus BREAST-Q	Breast augmentation	Prospective study	40 female participants. All patients were satisfied with the simulated outcomes representations. However, only the simulation of breast volume was very accurate.	Studies evaluating the efficacy of 3-D simulation regarding a pool of patients with breast ptosis should be conducted.

	Augmentation: A Prospective Study						
Pruksapong et al (2026), [28]	The Role of 3-dimensional Imaging and Telemedicine in Breast Augmentation Consultations: A Prospective Cohort Study of 1153 Cases and a Randomized Controlled Trial in Thailand	Plastic and Reconstructive Surgery Global Open - Thailand	3-D Simulation (Crisalix) and Telemedicine (online consultations via Line Call/Chat)	Breast augmentation	Prospective cohort study and randomized controlled trial	1153 participants in the cohort study, 120 participants in the randomized controlled trial. 3-D simulation facilitated individualized implant selection and prevented the development of unrealistic ecxpectations. The combination of telemedicine with 3-D simulation improved patient satisfaction and limited consultation time.	Further system refinements regarding patients with severe ptosis. This technology supports personalized patient-care and shared decision- making.
Ersan et al (2026), [5]	The impact of three-dimensional simulation and virtual reality technologies on surgical decision-making and postoperative satisfaction in aesthetic surgery: a preliminary study	Computer Assisted Surgery - Turkey	3-D Simulation (Crisalix Virtual Esthetics System)	Rhinoplasty, Breast augmentation, Mastopexy, Augmentation-mastopexy and Breast reduction	Retrospective observational study	75 female patients. 3D simulation influenced substantially decision-making in rhinoplasty, breast augmentation and augmentation-mastopexy patients.However, the most important factors in decision-making, according to patients, were patient-surgeon communication and photographic outcomes from other patients. Most patients would recommend choosing a clinic offering 3-D simulation in decision-making.	Larger cohorts should confirm the findings. Technology should accompany patient-surgeon communication rather than replace it.
Işıktekin et al (2026), [29]	An Evaluation of Breast Augmentation: Accuracy, Utility, and Accessibility	Aesthetic Plastic Surgery - Turkey	Large Language Models (ChatGPT, Google Gemini)	Breast augmentation	Observational study	10 plastic surgeons. ChatGPT presented better performance than Google Gemini in providing structured baseline	These platforms may assist physicians during patient consultation by providing basic, comprehensible

	of Knowledge for Patient Education and Consultation with Artificial Intelligence					information to patients' inquiries.	information prior to treatment planning, facilitating a more focused conversation.
Shiraishi et al (2024), [30]	Blepharoptosis Consultation with Artificial Intelligence: Aesthetic Surgery Advice and Counseling from Chat Generative Pre Trained Transformer (ChatGPT)	Aesthetic Plastic Surgery - Japan	Large Language Model (ChatGPT)	Upper Blepharoplasty	Observational study	4 plastic surgeons and 4 non-medical staff university members. ChatGPT may emulate patient-physician conversation and provide clear and comprehensible information, acknowledging also its constraints. Non-medical participants considered it more informative than surgeons.	Studies with larger samples are required as well as research regarding clinical benefits of the use of ChatGPT in routine practice.
Copeland-Halperin et al (2023), [31]	Evaluation of Artificial Intelligence–generated Responses to Common Plastic Surgery Questions	Plastic and Reconstructive Surgery Global Open - USA	Large Language Models (ChatGPT, Bing)	Plastic surgery	Exploratory study	2 plastic surgeons. The AI tools provided accurate information to general clinical inquiries but were inefficient in terms of decision-making issues.	Human oversight as well as algorithms should evalute and dissociate AI-generated information according to standards.
Seth et al (2023), [32]	Evaluating Chatbot Efficacy for Answering Frequently Asked Questions in Plastic Surgery: A ChatGPT Case Study Focused on Breast Augmentation	Aesthetic Surgery Journal - Australia	Large Language Model (ChatGPT)	Breast augmentation	Qualitative study	3 plastic surgeons. ChatGPT provided accurate information facilitating managing patients' expectations but lacked efficacy in queries for personalized advice and was considered inconsistent regarding the accuracy of references and sources.	The algorithms should be trained to prioritize evidence-based information and dissociate disreputable sources. Visual tools could also contribute to its capacity to comprehensively inform patients.
Zhang et al (2025), [33]	Potential of AI Chatbots in Online Hair Transplantation Consultations: A Multi-metric	Aesthetic Plastic Surgery - China	Large Language Models (ChatGPT, Claude, Gemini Advanced, Sonnet)	Hair transplantation	Qualitative study	3 hair transplantation surgeons. The chatbots demonstrated high performance in terms of accuracy of information,	Larger and more variable training datasets are necessary as well as constant monitoring to overcome

	Assessment of Three Models					understandability and empathy	privacy and liability challenges. Chatbots could provide initial information and complement the physician's consultation.
Radulesco et al (2025), [34]	Evaluation of Artificial Intelligence Chatbots for Facial Injection Planning: Comparative Performance and Safety Limitations	Aesthetic Plastic Surgery - France and Italy	Large Language Models (ChatGPTo1, ChatGPT4o, Gemini 2.0, Claude-3.5-Sonnet, Copilot Pro and Llama 3.3)	Injectable treatments (botulinum toxin and hyaluronic acid fillers)	Comparative observational study	40 patients, 2 otolaryngologists with training in aesthetic facial surgery. ChatGPT presented superior performance providing accurate protocols and standardized approaches. However, lack of personalization and safety omissions undermined its applicability.	Larger multi-center studies including comparison to human assessments are necessary to validate the findings. Development of models capable of generating personalized plans and safety protocols. AI should be utilized as an auxiliary tool in patient care.
Shao et al (2025), [35]	Evaluating artificial intelligence chatbots' responses to gynecomastia inquiries: Comparative study of information quality, readability, and guideline consistency	Digital Health - China	Large Language Models (ChatGPT, DeepSeek, Gemini, Perplexity, and Copilot)	Gynecomastia	Comparative study	2 breast surgeons. ChatGPT demonstrated the highest accuracy score with regard to common patient concerns. However, omissions concerning guideline details were noted and readability was variant across the AI chatbots.	Training data should be optimized and experts should oversee the function of algorithms and AI- generated content. A regulatory framework should also be established by healthcare authorities.
Brito et al (2024), [36]	Assessing ChatGPT's Reliability as an Educational Resource for Information on Breast Reduction Surgery	Cureus - USA	Large Language Model (ChatGPT)	Breast reduction	Observational study	5 plastic surgeons. ChatGPT was able to provide basic knowledge, however omissions of important details as well as inconsistencies were observed.	ChatGPT may facilitate a more productive and focused consultation, however it cannot replace the supervision of a human expert.
Bastaninejad et al (2026),	Evaluating the Insights of ChatGPT,	Aesthetic Plastic Surgery	Large Language Models (ChatGPT,	Revision rhinoplasty	Comparative study	4 otolaryngologists. AI chatbots provided accurate	AI developers should utilize evidence-based

[37]	Gemini and Expert Surgeons in Revision Rhinoplasty Consultation	- Iran	Gemini)			and empathetic information, which can be utilized prior to consultations with physicians.	resources and refine the personalization of AI-generated responses. Surveillance should be developed in terms of ethical standards. AI applications cannot replace the human assessment in complex cases.
Rivera et al (2026), [38]	Evaluating the Quality and Reliability of Large Language Models for Plastic Surgery Patient Education: A Comparative Analysis of ChatGPT and OpenEvidence	Aesthetic Surgery Journal - USA	Large Language Models (ChatGPT, OpenEvidence)	20 Aesthetic surgery procedures	Comparative study	2 plastic surgery research fellows. OpenEvidence provided information of higher quality and reliability as well as citations and references regarding its sources.	Larger studies should validate the findings, while OpenEvidence may complement the expert's consultation providing relevant information.
Abi-Rafeh et al (2024), [39]	Preoperative Patient Guidance and Education in Aesthetic Breast Plastic Surgery: A Novel Proposed Application of Artificial Intelligence Large Language Models	Aesthetic Surgery Journal Open Forum - Canada and USA	Large Language Model (ChatGPT)	Breast augmentation, Mastopexy, Breast reduction	Observational study	4 plastic surgeons. ChatGPT demonstrated acceptable levels of accuracy, comprehensiveness, safety and clarity, acknowledging its limitations. Omissions of important information such as risks and complications were also reported.	Large Language Models may serve as adjuncts to patients' education facilitating preoperative counseling, but they should not be considered a substitute for human expert judgment.
Yun et al (2023), [40]	A comprehensive evaluation of ChatGPT consultation quality for augmentation mammoplasty: A comparative analysis between plastic	International Journal of Medical Informatics – Republic of Korea	Large Language Model (ChatGPT)	Breast augmentation	Qualitative study	5 plastic surgeons and 5 laypersons evaluated the answers provided by ChatGPT. The scores provided by plastic surgeons were lower than the rating within laypersons in terms of reliability, quality of information and	The development of appropriate assessment tools is recommended for the evaluation of the quality of AI-derived information.

	surgeons and laypersons					emotional management.	
Bukret et al (2025), [41]	Artificial Intelligence–assisted Risk Stratification in Aesthetic Surgery: A Prospective Observational Study	Plastic and Reconstructive Surgery Global Open - Argentina	AI risk assessment model (Bukret AI-based risk assessment model)	Elective aesthetic surgeries	Prospective observational study	3347 patients (2886 female, 461 male). The AI risk assessment model facilitates decision-making by predicting complications and engendering tailored recommendations accordingly to mitigate them.	A randomized controlled trial with a control arm should be conducted, including demographic data and impact on patient satisfaction.
Basile et al (2024), [42]	Using Machine Learning to Select Breast Implant Volume	Plastic and Reconstructive Surgery - Brazil	Machine Learning predictive model	Breast augmentation	Retrospective study	1057 participants (800 for training, 200 for validation, 57 reoperation cases. The AI model demonstrated high precision in selecting the appropriate breast implant which may facilitate the decision-making process and improve patient satisfaction.	The author proposes the integration of the model into the daily practice as a decision support system to promote personalized care.
Bian et al (2025), [43]	Construction of a patient-centered decision support tool for cosmetic breast surgery	Chinese Journal of Plastic and Reconstructive Surgery - China and Malaysia	Patient-centered Decision Aid (PDA)	Breast augmentation and Breast reduction	Pilot study	12 focus group participants, 20 participants in the pilot study. The PDA facilitated the decision-making process by improving communication, clarifying patients' needs, providing comprehensible information and reducing decisional regret.	Multi-center trials with larger sized samples should be conducted to explore the tool's impact on quality of life.
Kolasiński et al (2025), [44]	The Effect of Patient Involvement in Implant Size Selection on Satisfaction With Breast Size: An Analysis of 1840	Aesthetic Surgery Journal - Poland	Patient's Decision-Making Process on Implant Size Selection (PIS) algorithm including 3-D simulation (Vectra 3D system)	Breast augmentation	Single-center retrospective Study	1840 patients. The rate of patient dissatisfaction with the outcome was less than 1%. Sizer use and 3-D simulation helped the patients make the final decision concerning implant	The findings should be validated by studies including more diverse populations.

Primary Augmentations	and external sizers	choice within a given range of options.
